# Linkage of historical GB Census data to present day population registers.

**DOI:** 10.23889/ijpds.v7i3.2002

**Published:** 2022-08-25

**Authors:** Paul Longley, Justin van Dijk, Tian Lan

**Affiliations:** 1 University College London

## Objectives

This research links, for the first time, individual level entire GB population census data for 1851-1911 to present day individual level population registers. Precise georeferencing of individual records using AddressBase Premium and further linkage to Indices of Multiple Deprivation (IMDs) enables family group analysis of inter-generational social mobility outcomes.

## Approach

Present-day individual names and addresses taken from Electoral Registers and consumer data are georeferenced and linked to IMD data using AddressBase. This enables calculation of average IMD scores for every family group (surname). Individual level names and addresses from 1851-1911 censuses are assigned to harmonised historical parishes. The present day surname IMD scores are attributed to every resident in the historical censuses. Parish average ‘future IMD’ scores are calculated to show which areas have bequeathed the highest and lowest IMD scores on their residents’ descendants. This is a measure of how ‘north – south divides’ shape inter-generational social mobility.

## Results

A linked data website, apps.cdrc.ac.uk/gbnames, profiles social mobility outcomes for 13,000+ family names, according to average neighbourhood quality experienced by family name bearers. There are clear and enduring regional divides in “future deprivation” inherited from ancestors by the present-day GB population. The research traces the origins of a north-south divide in England. Family roots in northern industrial cities are associated with unfavourable outcomes today. In Scotland, an east-west divide identifies eastern areas sharing similarly high levels of hardship to nineteenth-century industrial cities such as Liverpool and Manchester. Migration partially mitigates these inequalities, but most family groups remain concentrated in their ancestral heartlands, and continue to experience the long-term disadvantages bestowed by geographical location.

## Conclusion

Surnames provide an under-exploited way of linking precisely georeferenced geographies of entire historical populations to their descendants today. Additional linkage to areal deprivation measures makes it possible to evaluate how social and spatial inequalities both endure and are promulgated through the generations. Geography is destiny for much of the population.

